# Tensile Properties and Constitutive Model of BFRP–Steel–BFRP Composite Plates

**DOI:** 10.3390/ma18040756

**Published:** 2025-02-08

**Authors:** Yirui Zhang, Jiyang Yi, Yang Wei, Hu Zhao

**Affiliations:** 1 College of Civil Engineering, Nanjing Forestry University, Nanjing 210037, China; zyr94@njfu.edu.cn (Y.Z.); yjyaegean@njfu.edu.cn (J.Y.); 2 Jiangsu Highway Intelligent Detection and Low Carbon Maintenance Engineering Technology Research Center, Nanjing 210037, China; 3 Jiangsu GMV New Materials Technology Development Co., Ltd., Nanjing 210004, China; zhaohu08263917@sina.com

**Keywords:** FRP sheet, carbon steel plate, composite materials, tensile properties, constitutive model

## Abstract

Traditional materials such as steel and concrete often face limitations in terms of corrosion resistance and long-term performance. Over the past few decades, the search for alternative reinforcement solutions has grown, driven by the need for more sustainable, lightweight, and corrosion-resistant materials. Basalt fibers, with their superior mechanical properties and resistance to environmental degradation, have emerged as a promising candidate. This study investigated the tensile mechanical properties and constitutive behavior of basalt fiber-reinforced polymer (BFRP)–steel–BFRP composite plates. A total of 12 specimens were fabricated, varying in BFRP layer thickness, and subjected to uniaxial tensile testing. The results reveal that bonding BFRP layers significantly enhances the strengthening stiffness and strength of the steel plates, while maintaining ductility and fracture stability. The stress–strain analysis indicates a bilinear behavior, with the BFRP layers contributing to a higher slope during the strengthening stage and stable fracture strain across specimens. Additionally, a three-segment constitutive model was proposed and validated, demonstrating high accuracy in predicting tensile behavior. The findings highlight the potential of BFRP–steel–BFRP composite plates as efficient reinforcement solutions, offering a balance of strength, flexibility, and cost-effectiveness. This study provides data and modeling insights to guide the design and optimization of composite materials for structural applications.

## 1. Introduction

With the acceleration of urbanization, aging structures face significant safety risks due to material degradation and outdated design standards, making it challenging to meet current safety and usage demands. Strengthening techniques, as a key means of enhancing structural load-bearing capacity [1], durability [2], and seismic performance [3], can extend the service life of structures while avoiding resource wastage and environmental pollution caused by demolition and reconstruction. Combining advanced fiber-reinforced polymers (FRP) materials with traditional steel in reinforcement methods offers advantages such as high strength [4,5], lightweight properties [6,7], and ease of construction [8,9]. This approach provides an efficient and cost-effective solution for the renovation of aging structures [10,11], demonstrating significant engineering value and social importance.

**Figure 1:**
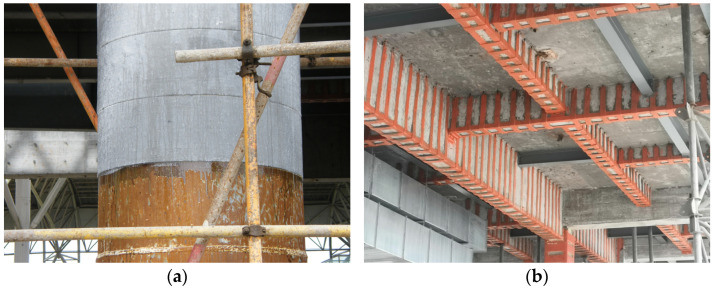
(**a**) FRP sheet reinforcement for the column; (**b**) FRP plate reinforcement for the beams.

Over the past few decades, carbon fiber-reinforced laminates (CFRPs) and glass fiber-reinforced laminates (GFRPs) have been extensively studied on existing structures’ reinforcement (Figure 1). Du et al. [12,13] proposed a method of strengthening steel structures using FRP plates with stiffening ribs. The study investigated the effects of this method on the static properties of steel plates and found that, after strengthening, the out-of-plane stability, shear stiffness, and load-carrying capacity of the steel plates were significantly improved, while fatigue damage was reduced. Du et al. [14] developed an FRP–steel composite plate (FSP) and analyzed its mechanical properties through cyclic tensile tests and theoretical modeling. The effective dimensions of the tested specimens are 200 × 30 mm, with the steel plate thickness ranging from 6 mm to 10 mm. The results stiffness indicated that the FSP exhibits a bilinear stress–strain relationship, more stable post-yield, and reduced residual deformation, outperforming traditional steel plates. Zheng et al. [15] investigated the mechanical behavior of FSPs under uniaxial static tensile loading. The effective dimensions of the tested specimens are 200 × 30 mm, with the steel plate thickness ranging from 5.73 mm to 9.63 mm. Through a combination of experiments, theoretical modeling, and finite element analysis, they revealed the bilinear stress–strain characteristics and failure modes of the composite plates. Wang et al. [16] and Hu et al. [17] also explored the effects of FRP on improving the fatigue performance of cracked steel plates through experimental and numerical analysis. The results show that CFRP significantly reduces crack propagation rates, extending the fatigue life of centrally cracked specimens by 3.5–4.9 times and edge-cracked specimens by 6.9–11.3 times. Nhut and Matsumoto [18] investigated the reinforcing effects of CFRP on thin steel plates under shear loading through experiments, theoretical modeling, and finite element analysis. They evaluated two CFRP fiber orientations (0°/90° and ±45°), demonstrating a significant improvement in shear strength. Li et al. [19] proposed a novel basalt fiber reinforced polymer (BFRP)–steel composite plate (BSP) and experimentally studied its mechanical behavior under uniaxial tensile and cyclic tensile loading. The effective dimensions of the tested specimens are 130 × 25 mm, and the steel plate thickness is 3.05 mm. The results showed that the BSP exhibits bilinear stress–strain characteristics, stable post-yield stiffness, and increased ultimate load-bearing capacity with the addition of more BFRP layers, along with minimal residual deformation after yielding.

Existing studies [14,19] have demonstrated that different types of FRP materials exhibit excellent performance and significant effectiveness in steel plate reinforcement, as shown in Table 1. Among them, CFRP, due to its superior stiffness and strength, can significantly enhance the initial stiffness and yield strength of steel plates, while GFRP and BFRP offer advantages in improving ductility.

Additionally, the number of FRP layers and their form (e.g., sheets or plates) have garnered considerable attention for their impact on reinforcement performance. Increasing the number of FRP layers can significantly improve the yield strength and stiffness of steel plates; however, the marginal benefits of additional layers gradually diminish. Notably, CFRP plates are particularly effective in enhancing the peak strength of steel plates. These findings indicate that the type, number, and form of FRP are critical factors in determining the performance of steel plate reinforcement.

Previous studies have demonstrated the excellent mechanical performance of FSPs under tensile loading conditions. Despite these advantages, the high cost of carbon fiber and the relatively lower performance of glass fiber limit their broader application in cost-sensitive areas. Basalt fiber, as an emerging natural fiber-reinforced material, has garnered increasing attention due to its excellent mechanical properties [20,21], chemical durability [2,22,23], and cost-effectiveness [24]. Compared to carbon fiber and glass fiber, basalt fiber offers higher tensile strength, superior thermal stability, and environmental sustainability.

However, despite these advantages, research data on BSP remain relatively limited, with most studies focusing on the unilateral reinforcement of steel plates. The lack of understanding of the tensile response and failure mechanisms of BSPs has hindered their practical application in structural design and reinforcement.

This study systematically investigates the tensile mechanical properties of basalt fiber–steel composite plates using a combination of experimental and modeling approaches. Tensile tests are conducted to characterize the material’s strength, stiffness, and failure modes, while calculation models are proposed to predict its tensile behavior and analyze the interaction mechanisms between the basalt fiber and steel layers. The findings are expected to provide valuable reference data for the design and optimization of BFRP–steel composite structures.

## 2. Specimen Design and Preparation

### 2.1. Details of Specimen

This study proposed a novel composite plate structure aimed at investigating its monotonic axial tensile performance and corresponding models. Therefore, the experimental design did not account for the effects of bond-slip behavior. The intended application scenarios for the proposed composite plates are based on cases with anchorage at both ends, such as in prestressed systems.

The experiment designed four groups of parameters, with a total of 12 specimens (Table 2). BF0 consists of pure steel specimens, serving as a baseline for comparing the strengthening effect of bonded BFRP sheets. BF2, BF4, and BF6 represent BFRP–steel–BFRP composite plates (BSBCPs) bonded with two, four, and six layers of BFRP sheets, respectively. The BFRP sheets were symmetrically bonded on both sides of the steel plate. Thus, the number of layers bonded on each side corresponds to one, two, and three layers, respectively. Three identical specimens were designed for each parameter group to evaluate the discreteness and reliability of the test results.

**Figure 2:**
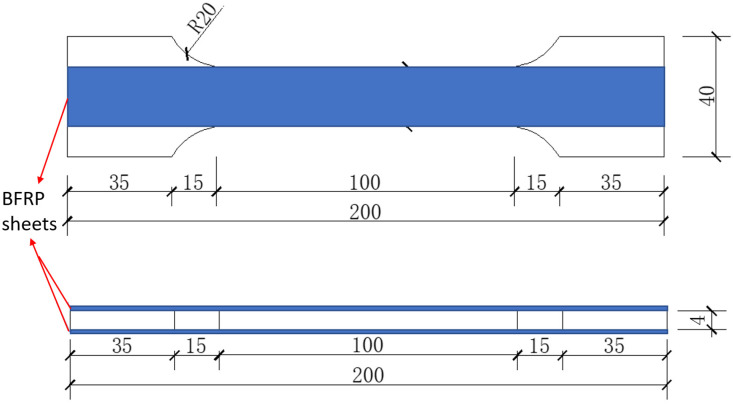
Dimensional details of composite plates (unit: mm).

Figure 2 presents the specific dimensional parameters of the composite plates. The thickness of the steel plate for all specimens is uniformly 4 mm. The dimensions of the steel plate were determined in accordance with the standard ISO 6892-1:2019 [25]. The BFRP sheets are rectangular strips measuring 200 × 20 mm and are symmetrically bonded to both sides of the steel plate.

### 2.2. Preparation Process

**Figure 3:**
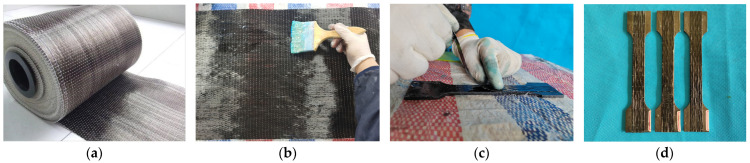
(**a**) Raw BFRP sheets. (**b**) Impregnation of sheets. (**c**) Pasting of sheets. (**d**) BFRP–steel–BFRP composite plates.

Figure 3 illustrates the preparation process of the composite plates. First, dry fabric of appropriate dimensions is cut from the BFRP roll (Figure 3a). The surface of the steel plate is then cleaned to remove dust and rust. The BFRP fabric is fully impregnated with epoxy resin adhesive (Figure 3b). The impregnated BFRP fabric is accurately bonded to both sides of the steel plate (Figure 3c). For specimens with multiple layers of BFRP, this process is repeated. After 48 h, the epoxy resin adhesive cures, completing the preparation of the specimen (Figure 3d).

## 3. Material Properties and Test Layout

**Figure 4:**
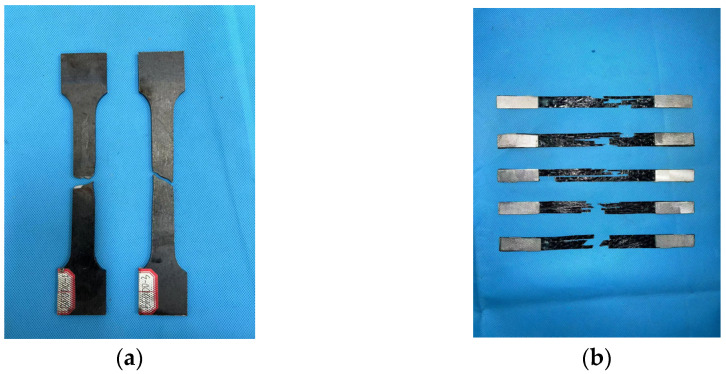
(**a**) Tensile test for steel. (**b**) Tensile test for BFRP sheet.

The Q345B steel plates used were laser-cut from the same batch of steel material. The measured yield strength of the steel is 354.3 MPa; the yield strain is 0.172%, and the elastic modulus is 206.1 GPa (Figure 4a). The nominal thickness of a single-layer BFRP sheet is 0.114 mm. The tensile properties of the BFRP sheets were tested in accordance with ASTM D3039/D3039M [26] (Figure 4b). A total of eight standard tensile specimens were tested, and the average tensile property values obtained are as follows: tensile strength of 1752.1 MPa, ultimate tensile strain of 2.31%, and elastic modulus of 75.3 GPa. The epoxy resin used (L500-AS/L-500BS) was produced by Nanjing Mankate Technology Co., Ltd. (Nanjing, China). Its mechanical properties were provided by the manufacturer: ultimate tensile strength of 62.4 MPa, elastic modulus of 3.4 GPa, and ultimate tensile strain of 0.02.

**Figure 5:**
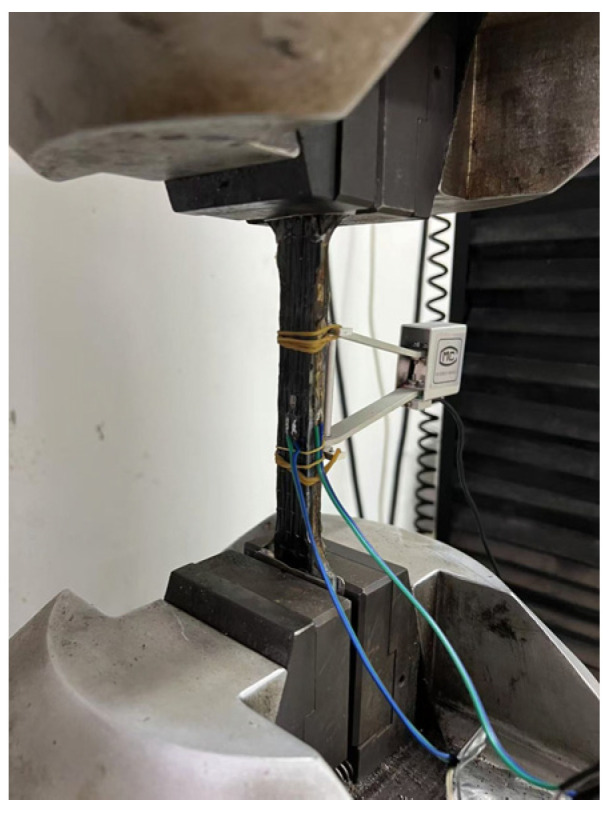
Test layout.

Figure 5 illustrates the testing method for the specimens. The tensile performance was evaluated using a universal testing machine at a loading rate of 0.2 mm/min. An extensometer with a gauge length of 50 mm and two strain gauges were installed at the center of the composite plate to measure strain over different gauge lengths. The tensile strain was obtained by cross-verifying the data from the two devices. The testing load and displacement measured by the extensometer were directly recorded by the testing machine, while the strain gauge data were collected using an external data acquisition system.

## 4. Results Discussion

### 4.1. Failure Modes

The tensile failure mode of the BF0 series specimens is shown in Figure 4a. The failure of the steel occurred through necking and ductile fracture, characterized by a localized reduction in cross-sectional area (necking zone). The fracture surface exhibited a fibrous appearance. This behavior is consistent with the findings of most previous studies [27,28,29].

**Figure 6:**
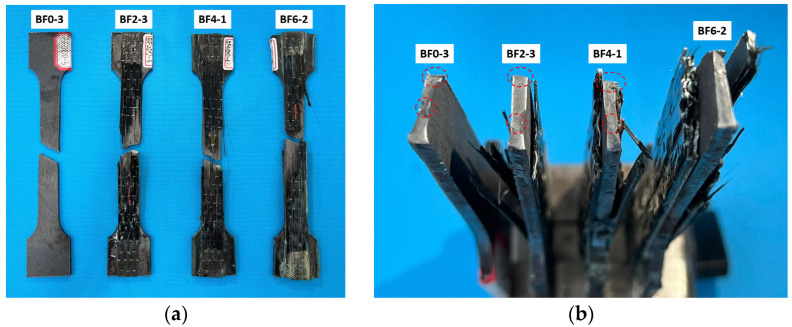
(**a**) The frontal view of the failed specimens. (**b**) The cross-sectional surface.

Figure 6 presents the failure modes of the four series of specimens. It can be observed that the composite plates exhibited similar failure behaviors. The fracture locations of both the steel and BFRP sheets predominantly occurred at the center of the specimens, with no failure observed at the ends. The good bonding between the end regions of the BFRP sheets and the steel plates indicates that the strengths of both materials were fully utilized.

Due to the significant difference in the tensile elastic modulus between the BFRP and steel, synchronized deformation throughout the loading process could not be maintained. As a result, debonding occurred at the center of the specimens between the BFRP sheets and the steel, which is an unavoidable issue [12,15,30]. Ultimately, the steel still exhibited a necking zone, characterized by a smaller cross-sectional area in the center compared to the sides. The fracture surface of the BFRP sheets was not horizontal but displayed an inclined cutting plane.

**Figure 7:**
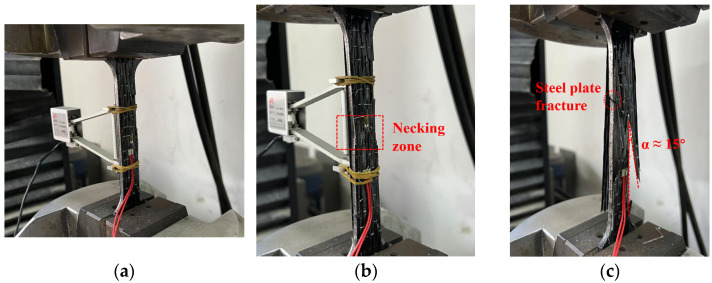
(**a**) Initial loading phase of BF2-2. (**b**) Middle loading phase of BF2-2. (**c**) Later loading phase of BF2-2.

Figure 7 illustrates the complete failure process of the BF2-2 specimen. Figure 7a shows the early loading stage of the composite plate. It can be observed that, during the initial elastic loading phase, no significant damage occurred, and no delamination appeared between the BFRP sheets and the steel plate.

As the load increased, a noticeable necking zone developed at the center of the composite plate (Figure 7b). Both the steel plate and the BFRP sheets exhibited a significant reduction in cross-sectional area, indicating that the composite plate had entered the plastic loading phase.

Finally, Figure 7c presents the ultimate failure mode of the composite plate. It is evident that delamination occurred between the BFRP sheets and the steel plate. The sandwich steel plate fractured near the upper part of the central region, while the fracture surface of the BFRP sheets exhibited an inclination of approximately 15°. The fracture locations on both sides occurred at the center and the ends of the specimen, respectively.

### 4.2. Stress–Strain Curves

**Figure 8:**
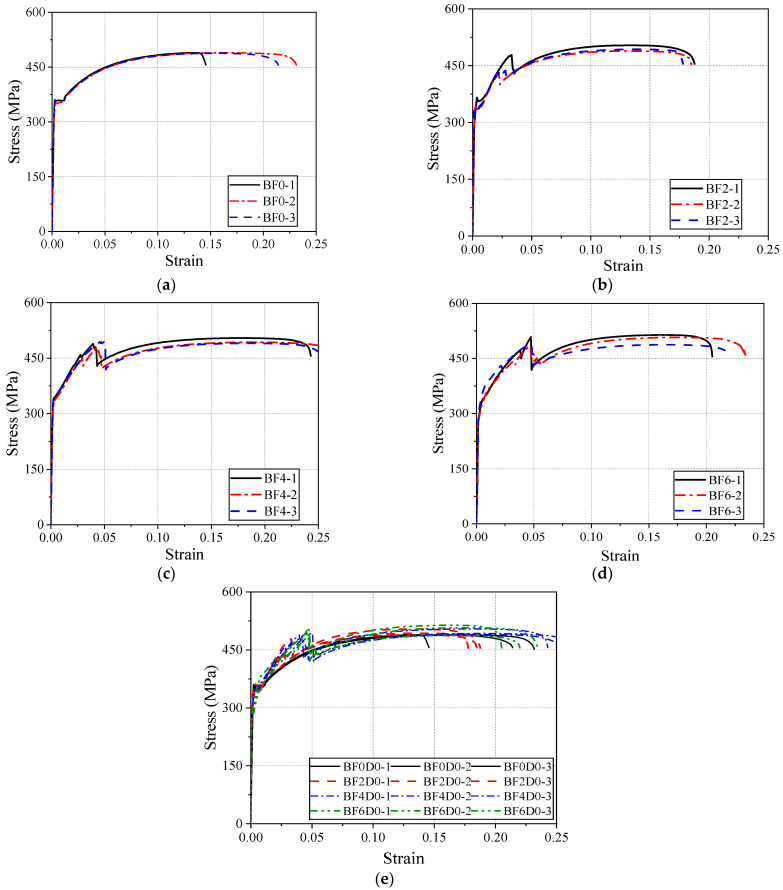
(**a**) BF0 series; (**b**) BF2 series; (**c**) BF4 series; (**d**) BF6 series; (**e**) all series.

Figure 8 presents the stress–strain curves for four series of specimens. Figure 8a shows the typical tensile constitutive relationship of steel. The stress–strain curves of the steel exhibit four distinct stages: the elastic stage, yield stage, strengthening stage, and necking stage. In the elastic stage, stress is proportional to strain; during the yield stage, a stress plateau appears with fluctuations; in the strengthening stage, stress increases, leading to significant plastic deformation; and, in the necking stage, a reduction in local cross-sectional area ultimately leads to fracture. The three specimens exhibit similar tensile behavior, indicating that the material strength of the steel has low variability.

Figure 8b shows the stress–strain curves of the steel plate with a single layer of BFRP bonded to each side. It can be observed that the reinforcement provided by the BFRP sheet shortens the length of the yield plateau and increases the yield slope, indicating that the BFRP significantly affects the yield behavior of the steel. In the strengthening stage, due to the high strength and linear elastic characteristics of the BFRP, the stress–strain curves of the composite plates show a higher slope, demonstrating a significant enhancement in the tensile stiffness of the composite material. As the tensile load progresses, the BFRP sheet eventually fractures, causing a sharp drop in tensile force, and the steel plate then returns to a state of independent tension. Thus, during the necking stage, there is no significant difference in the tensile behavior between the composite plate and the steel plate.

Figure 8c,d shows the stress–strain curves of specimens with two and three layers of BFRP sheets bonded to each side of the steel plate, respectively. Compared to the BF2 series, no significant differences are observed in the elastic stage for the BF4 and BF6 series. The yield section transitions from a plateau to a smooth, sloping curve, presenting a bilinear characteristic. In the strengthening stage, all three series exhibit a similar secondary strengthening trend, and, as the number of BFRP layers increases, the slope of the strengthening section remains unchanged. However, the fracture strain is significantly increased (see Figure 8e). Therefore, the maximum stress of the composite plates is increased in the strengthening section, but the maximum tensile stress during the necking stage is similar for all specimens.

**Figure 9:**
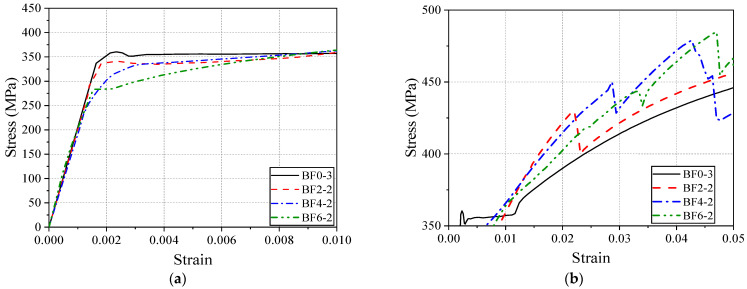
(**a**) Zoomed-in view of the yield stage. (**b**) Zoomed-in view of the strengthening stage.

To further analyze the effects of BFRP sheet bonding on the yield and strengthening stages of the specimens, Figure 9 provides a magnified view of these two phases.

According to the data in Figure 9a, the elastic stiffness of the BF0 (pure steel), BF2-2, BF4-2, and BF6-2 specimens is 206.1 GPa, 201.0 GPa, 192.3 GPa, and 193.8 GPa, respectively. This indicates that bonding two, four, and six layers of BFRP sheets decreased the elastic equivalent stiffness of the reinforced specimens by 2.5%, 6.7%, and 6.0%, respectively, compared to the pure steel specimen.

Figure 9b magnifies the slope of the strengthening stage curve. To avoid the influence of BFRP fracture, the slope is calculated for the strain range of 0.025–0.035. The strengthening stiffness of BF0, BF2-2, BF4-2, and BF6-2 is 1772.9 MPa, 2050.0 MPa, 2215.8 MPa, and 2837.1 MPa, respectively. This indicates that bonding two, four, and six layers of BFRP sheets increased the strengthening stiffness by 15.6%, 25.0%, and 60.0%, respectively, compared to pure steel.

Overall, compared to the original steel plate, bonding BFRP primarily affects the yield and strengthening stages of the steel, with minimal impact on the elastic and necking stages. This result is significant for the design of composite structures. On the one hand, BFRP effectively suppresses the yielding deformation of steel; on the other hand, even if steel yields, the composite plate still provides significant tensile reserve capacity. Additionally, increasing the number of BFRP layers can improve the stability of the fracture strain.

### 4.3. Analysis of Key Parameters

#### 4.3.1. Yield Point

**Figure 10:**
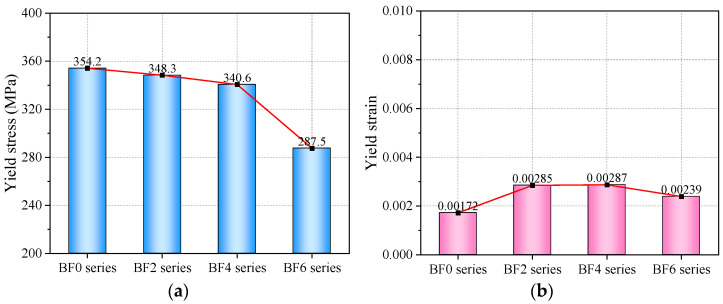
(**a**) Yield stress. (**b**) Yield strain.

Figure 10 presents the yield-point data for each series of specimens. For reinforced specimens, the yield stage is represented by a transitional curve. The method for determining the yield point is to select the endpoint of the transitional curve, which corresponds to the starting point of the linear strengthening stage. From Figure 10a, it can be observed that, as the number of BFRP layers bonded to both sides of the steel plate increases, the yield stress shows a decrease of 1.7–18.8%. This is likely because, at the yield stage of the composite plate, both the BFRP and the steel share the load, but the relatively low elastic modulus of the BFRP sheets prevents them from fully exhibiting their high-strength characteristics at low yield strains. In this case, the BFRP sheets cannot exceed the yield strength of the steel, thereby weakening the composite plate’s overall yield strength. However, the yield strain exhibited a slight increase. The yield strain of the BF6 series is 1.39 times that of the BF0 series. This indicates that, when a sufficient number of BFRP sheets are bonded, the yield deformation capacity of the steel plate can be enhanced, but the yield stress will decrease. This finding is crucial for the performance design of composite material plates, making them suitable for structures that require greater deformation capacity. This will determine the serviceability limit state of composite plates.

#### 4.3.2. BFRP Fracture Point

Based on the statistical analysis of all data, it was found that the tensile stress at the peak of the strengthening stage (i.e., the BFRP fracture point) is very close to the tensile stress at the peak of the necking stage. Therefore, the analysis of the BFRP fracture stress in this section corresponds to the maximum tensile stress of the composite plate. After the fracture of the BFRP, the tensile stress of the reinforced specimens decreased by 35.1 MPa (BF2 series), 60.5 MPa (BF4 series), and 67.6 MPa (BF6 series), respectively. At this point, the tensile stress of the reinforced specimens was slightly lower than that of the BF0 series but gradually returned to the tensile stress level of pure steel as deformation increased. Thus, compared to the BF0 series, the addition of BFRP layers results in a sharp instantaneous drop in the stress–strain curve at the BFRP fracture point, but it quickly returns to the tensile behavior of the metal (Figure 8e).

**Figure 11:**
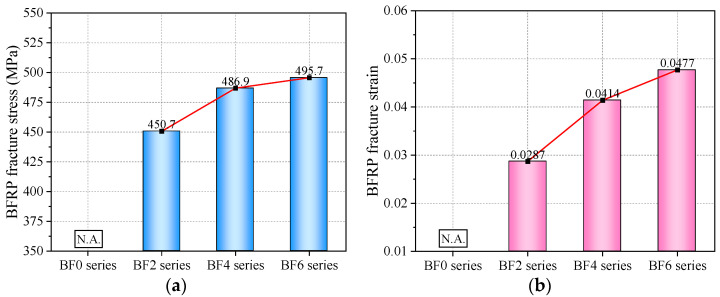
(**a**) BFRP fracture stress; (**b**) BFRP fracture strain.

Figure 11 compares the stress and strain of the reinforced specimens at the BFRP fracture point. From Figure 11a, it can be observed that the addition of four layers of BFRP sheets has a more pronounced effect on increasing the stress of the steel plate. The BFRP fracture stress increased from 450.7 MPa (BF2 series) to 486.9 MPa (BF4 series), representing an 8% increase. When comparing the BF6 series to the BF4 series, the increase was approximately 2%. This indicates that using BFRP sheets to reinforce the tensile performance of steel plates does not lead to a proportional increase in tensile stress with the number of layers. There is a nonlinear relationship between the number of reinforcement layers and the tensile stress. This is primarily because as the number of BFRP layers increases, interlayer slip between adjacent sheets becomes more likely, preventing coordinated deformation and causing some BFRP sheets to fracture first.

In contrast to stress, the BFRP fracture strain exhibits an approximately linear relationship with the number of BFRP reinforcement layers (Figure 11b), which is similar to the trend observed for the yield strain. However, due to the limited sample number in this study, the fracture strain may not necessarily continue to increase linearly as the number of BFRP sheet layers increases. This study analyzes only a finite set of sample data, and the observed trend requires further investigation through additional research.

## 5. Full Stress–Strain Curve Model

**Figure 12:**
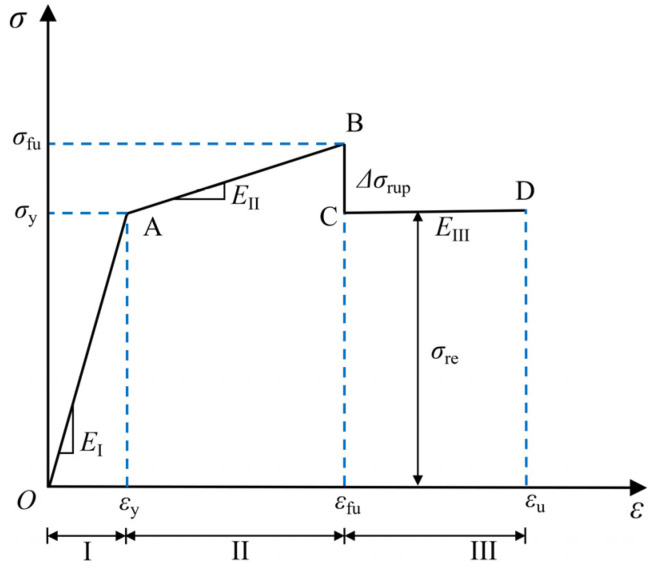
Tensile constitutive model of BSBCP.

The tensile behavior of FRP–steel composite plates (BSBCPs) has been studied by several researchers [14,19]. The theoretical model refers to the constitutive model of the steel–FRP composite bar (SFCB) [31]. The structural feature of SFCB is the steel bar wrapped with FRP, which is similar to the BSBCP discussed in this paper. Therefore, the theoretical results of SFCB research are also applicable to BSBCPs. In this section, the stress–strain curve of the BSBCP is modified based on the three-segment model proposed by Wu et al. [31]. Figure 12 shows the three stages of the improved model: the elastic segment, the strengthening segment, and the residual segment. The model in this paper primarily corrects the strain calculation method at two critical points (B and C) and further optimizes the residual segment.

Elastic Segment OA: The stiffness in this stage is the composite tensile stiffness of the steel and BFRP, denoted as E_I_, which can be expressed by Equation (1). ε_y_ represents the yield strain of the BSBCP. In Wu et al.’s model, the yield strain of steel is directly used for the calculation. ε_y_ is the yield strain of the steel, namely, 0.172% in this paper. The yield stress σ_y_ can be calculated using Equation (2).

E_I_ = (E_s_A_s_ + E_f_A_f_)/A, 0 ≤ ε ≤ ε_y_ (1)

σ_I_ = ε(E_s_A_s_ + E_f_A_f_)/A, 0 ≤ ε ≤ ε_y_ (2)

where E_s_ and E_f_ are the Young’s moduli of steel and BFRP, respectively; A_s_, A_f_, and A are the cross-sectional areas of steel, BFRP, and total section, respectively.

Strengthening Segment AB: The stiffness E_II_ in this stage is provided by the BFRP sheets, as expressed by Equation (3). ε_fu_ is the fracture strain of the BSBCP. Previous models did not consider the effect of the number of BFRP layers on the fracture strain of the composite plate. However, based on the findings of this study (Figure 11b), an increase in the number of BFRP layers leads to a linear increase in the fracture strain. Therefore, ε_fu_ is calculated using Equation (4). The fracture stress σ_fu_ can then be computed using Equations (3) and (4), as shown in Equation (5).

E_II_ = (E_f_A_f_)/A, ε_y_ ≤ ε ≤ ε_fu_ (3)

ε_fu_ = ε_f_ + 0.002n_f_, ε_y_ ≤ ε≤ε_fu_ (4)

σ_II_ = (f_y_A_s_ + εE_f_A_f_)/A, ε_y_ ≤ ε ≤ ε_fu_ (5)

where ε_f_ is the fracture strain of a single layer of BFRP, with the data in this study being 0.023; n_f_ is the number of BFRP layers applied; f_y_ is the yield stress of steel, which is taken as 354.3 MPa in this paper.

Residual Segment CD: After the BFRP fracture, the stress at point B drops sharply to point C and then keeps constant until the steel fracture point D. Therefore, the slope of the curve in segment CD is zero. The CD segment is essentially the necking stage of the steel. The stress loss due to BFRP fracture, Δσ_rup_, can be calculated using Equation (6). The coefficient in the formula is derived from a statistical regression of the stress loss observed after the fracture of different numbers of BFRP layers. When the number of BFRP layers increases from 1 to 3, the corresponding stress losses are 35.1 MPa, 60.5 MPa, and 67.6 MPa, respectively. It is important to note that, due to the limitations of the sample characteristics in this study, Equation (6) is only applicable to BFRP types. This allows for the determination of the location of point C. The position of point D can be determined using the steel fracture strain ε_u_.

Δσ_rup_ = 13.0n_f_, ε_fu_ ≤ ε ≤ ε_u_ (6)

**Figure 13:**
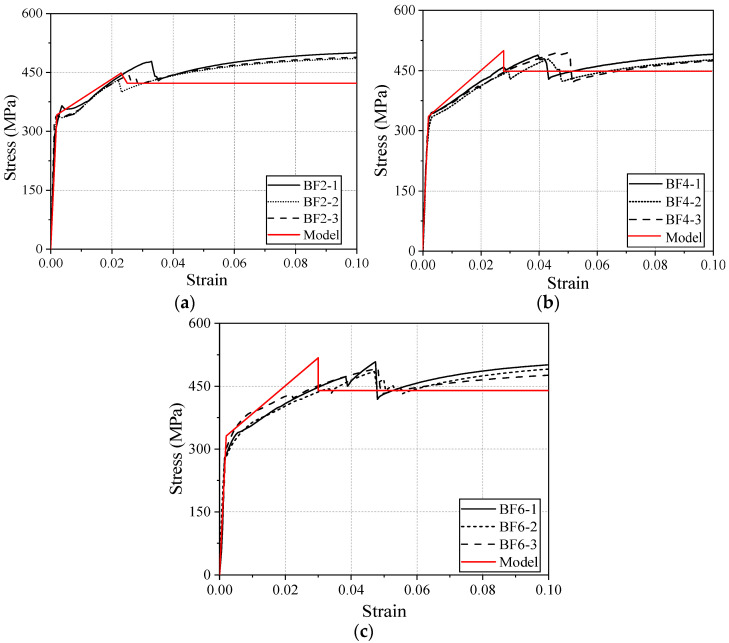
(**a**) Evaluations for BF2 series. (**b**) Evaluations for BF4 series. (**c**) Evaluations for BF6 series.

Figure 13 presents a comparison between the experimental data and the calculated stress–strain relationships for the BF2 series (a), BF4 series (b), and BF6 series (c), aiming to evaluate the accuracy and applicability of the model’s predictions. As shown in the figure, the model is able to roughly describe the material’s initial linear response behavior. In the post-yield strengthening phase and the residual phase, the predicted data align with the overall trend of the experimental data. However, some deviations are observed in specific regions, particularly near the peak stress and the yield point. The experimental data for the BF2 series show good consistency, and the model’s predictions are most accurate for this series; the BF4 and BF6 series exhibit slightly increased variability, with the model showing deviations in localized regions. These discrepancies are primarily attributed to the inherent variability of the BFRP material, leading to unstable fracture strains. Overall, the model demonstrates high applicability in describing the elastic and plastic behavior of BSBCPs.

## 6. Conclusions

This study systematically investigated the tensile performance and constitutive behavior of BFRP–steel–BFRP composite plates, providing valuable insights into their application in structural reinforcement. The results demonstrate significant improvements in tensile performance and propose a validated predictive model for mechanical behavior, aiding the practical design and optimization of such composite materials. The conclusions are summarized as follows:

(1) Bonding BFRP sheets to steel plates significantly enhances the strengthening stiffness and yield strength while maintaining ductility. The composite plates exhibit a bilinear stress–strain relationship with distinct strengthening effects in the post-yield stage.
(2) The fracture strain of the composite plates remains stable across specimens, and the addition of BFRP layers improves fracture stability. Failure modes indicate effective utilization of both steel and BFRP materials, with no premature debonding observed.
(3) The proposed three-segment constitutive model accurately predicts the tensile behavior of composite plates, including the elastic, strengthening, and residual stages. The model demonstrates high reliability and practicality.
(4) This study reveals a nonlinear relationship between the number of BFRP layers and tensile stress improvement. However, under compressive loads, the deformation and failure mechanisms of composites differ significantly from those of metals. Future research is needed to explore the compressive response and bonding behavior of hybrid composite systems.

## Figures and Tables

**Table 1:** The reinforcement effects of different types of FRPs on the steel plate.

Ref	Specimen	FRP Type	FRP Layers	Nominal Thickness of Steel Plate (mm)	Yield Strength (MPa)	Peak Strength (MPa)	Peak Strain (%)
[14]	FHA6-C1	CFRP sheet	1	6	312.6	356.5	1.05
[14]	FHA6-2C1	CFRP sheet	2	6	300.2	430.7	1.11
[14]	FHA6-3C1	CFRP sheet	3	6	296.2	429.3	1.08
[14]	FHA6-G1	GFRP sheet	1	6	304.9	340.5	1.22
[14]	FHA6-CP1	CFRP plate	1	8	271.9	559	1.57
[14]	FHA8-C1	CFRP sheet	1	8	323.4	371	0.98
[14]	FHA8-G1	GFRP sheet	1	8	329.8	382.9	1.45
[14]	FHA8-3G1	GFRP sheet	3	8	306.6	415.4	1.64
[14]	FHA8-5G1	GFRP sheet	5	8	273.5	402.3	1.52
[14]	FHA8-CP1	CFRP plate	1	8	274.2	602.3	1.78
[14]	FHA10-C1	CFRP sheet	1	10	358	359.6	0.96
[14]	FHA10-G1	GFRP sheet	1	10	325.2	382.4	1.69
[14]	FHA10-CP1	CFRP plate	1	10	279	651.5	4.25
[14]	HA6-1	-	-	6	315.1	-	-
[14]	HA8-1	-	-	8	309.5	-	-
[14]	HA10-1	-	-	10	316	-	-
[19]	BSP2	BFRP sheet	2	3.05	309.4	455.2	2.75
[19]	BSP4	BFRP sheet	4	3.05	293	483.8	2.48
[19]	BSP6	BFRP sheet	6	3.05	275.6	492	2.25
[19]	BSP8	BFRP sheet	8	3.05	258.6	551.2	2.51

**Table 2:** Specimen design.

Specimen No.	Steel Thickness (mm)	Layer of BFRP Sheet	Number of Composite Plates
BF0	4.0	0	3
BF2	4.0	2	3
BF4	4.0	4	3
BF6	4.0	6	3

## Data Availability

The original contributions presented in this study are included in the article. Further inquiries can be directed to the corresponding author.
